# A High-Durability Graphitic Black Pearl Supported Pt Catalyst for a Proton Exchange Membrane Fuel Cell Stack

**DOI:** 10.3390/membranes12030301

**Published:** 2022-03-07

**Authors:** Bing Li, Meng Xie, Kechuang Wan, Xiaolei Wang, Daijun Yang, Zhikun Liu, Tiankuo Chu, Pingwen Ming, Cunman Zhang

**Affiliations:** 1 Clean Energy Automotive Engineering Center, School of Automotive Studies, Tongji University, Shanghai 201804, China; xiemeng@tongji.edu.cn (M.X.); 2011672@tongji.edu.cn (K.W.); yangdaijun@tongji.edu.cn (D.Y.); liuzhikun@tongji.edu.cn (Z.L.); 1911679@tongji.edu.cn (T.C.); pwming@tongji.edu.cn (P.M.); zhangcunman@tongji.edu.cn (C.Z.); 2 Shanghai Composites Science & Technology Co., Ltd., Shanghai 201114, China; wangxiaolei7777@163.com

**Keywords:** Pt/graphitized black pearl (GBP) catalyst, carbon support, dynamic load cycle, durability, proton exchange membrane fuel cell

## Abstract

Graphitized black pearl (GBP) 2000 supported Pt nanoparticle catalysts is synthesized by a formic acid reduction method. The results of a half-cell accelerated degradation test (ADT) of two protocols and a single-cell ADT show that, Pt/GBP catalyst has excellent stability and durability compared with commercial Pt/C. Especially, the survival time of Pt/GBP-membrane electrode assembly (MEA) reaches 205 min, indicating that it has better reversal tolerance. After the 1003-hour durability test, the proton exchange membrane fuel cell (PEMFC) stack with Pt/GBP presents a slow voltage degradation rate of 5.19% and 36 μV h^−1^ at 1000 mA cm^−2^. The durability of the stack is improved because of the durability and stability of the catalyst. In addition, the post morphology characterizations indicate that the structure and particle size of the Pt/GBP catalyst remain unchanged during the dynamic testing protocol, implying its better stability under dynamic load cycles. Therefore, Pt/GBP is a valuable and promising catalyst for PEMFC, and considered as an alternative to classical Pt/C.

## 1. Introduction

The proton exchange membrane fuel cell (PEMFC) has become the most potential energy resource conversion device for its high-power density, zero emission and high energy conversion efficiency [1,2]. Although many achievements have been made in recent years, the cost and durability of FC stack are still the major hinders to large-scale applications of PEMFCs [3]. According to the estimation of the U.S. Department of Energy (DOE), the catalyst cost accounts for more than 40% of the whole stack. However, the stack lifetime is only about 4000 h, which is far less than the target of 8000 h [4].

Under actual working conditions, the stack performance decreases gradually due to the decay of various components or materials [5], including proton exchange membrane (PEM), catalyst, gas diffusion layer (GDL) and bipolar plate (BPP) [6,7,8]. Due to the important role in the stack, membrane electrode assembly (MEA) is usually called the FC stack’s heart. The three-phase boundary (TPB) composed of a catalyst, ionomer and reaction gas is the place where the electrochemical reaction takes place in the stack (i.e., the reactive active point). As the most important material of MEA, the durability of catalyst plays an important role in the lifetime of the stack [9], and further research is needed. Although great breakthroughs have been made in non-Pt catalysts [10,11,12,13], Pt is still the first choice for commercial FC applications due to its excellent electrocatalytic activity [14,15,16]. The main degradation mechanism of a supported Pt-based catalyst can be attributed to the growth and agglomeration of Pt nanoparticle, the corrosion of carbon support, and the weakening of the interaction between Pt nanoparticles and supports [17,18,19,20].

In terms of the control of Pt nanoparticles, Zhang [15] et al. proposed the strategy of molecular layer deposition (MLD) to stabilize Pt nanoparticles, which is conducive to the anchoring of Pt nanoparticles, avoid the catalyst degradation caused by particle agglomeration or desorption, improve the electron transfer between Pt nanoparticles and support, and improve the activity of the Pt nanoparticle catalyst. Beermann [21] found that the migration of Pt surface atoms would destroy the morphology of Pt-Ni nanoparticles, and seriously affected the materials’ electrocatalytic performance. The Pt-Ni octahedral nanoparticles had completely lost their octahedral shape after 8 k of potential cycles, but the Pt-Ni octahedral nanoparticles were doped with 3 at. % Rh still maintained their original morphology after 30 k of potential cycles under the same potential window, which implied that it had good stability.

On the other hand, although various types of supports have been used for FC catalysts, such as Ti-nanotube [22], SiC [23], SiO_2_ [24], TiO_2_ [1], Ta-doped Ti-oxide [25], hybrid material [26], etc., carbon materials are still the first choice for commercial catalyst supports. The properties of carbon support affect the uniform distribution, particle size, electrochemical active surface area (ECSA) and stability of nanoparticles, which have an important impact on the performance and durability of PEMFCs catalysts [27]. At present, various types of carbon supports have been used in FC, including graphite [28], graphene [29], activated carbon [30], carbon nanofibers [31], and carbon nanotubes [32,33]. Commercial Vulcan XC-72 carbon black has been widely used in FC ORR [34,35,36]. Although its proper specific surface area and porous structure are beneficial to improve the activity of ORR effectively, its low thermal and electrochemical stability are the main reasons for the rapid degradation of catalysis performance [37]. Carbon corrosion could lead to the detachment, and agglomeration of catalyst nanoparticles, causing the reduction of ECSA, of the thinning of catalytic layer thickness, and thus reducing of the performance and durability for PEMFCs [38,39,40,41]. The causes of carbon corrosion include a high temperature, high humidity, high-potential environment, high oxygen concentration, and fuel starvation in the FC stack [40,41,42]. Chung et al. [14] reported that graphitized carbon supported Pt nanoparticles were used as high efficiency FC catalysts, and found that the destruction of support pore structure and the agglomeration of Pt catalysts led to the loss of catalyst performance. To solve these problems, two different graphitization methods, thermal graphitization and catalytic graphitization, were used to treat the catalyst to obtain a unique porous structure, improving the utilization of Pt nanoparticles. Zhao et al. [43] prepared the Pt-based catalysts on Vulcan XC-72 and Ketjenblack EC 600-JD as supports. The above carbon materials were heat-treated at 1100 °C, 1600 °C and 2000 °C, respectively. The results showed that the higher graphitization degree could effectively improve the high-potential corrosion resistance of carbon support, enhancing the electrochemical stability of the catalyst. However, the anchoring ability of nanoparticles on the carbon supports will be reduced simultaneously. The carbon support treated at 1600 °C showed the highest durability, indicating that the graphitization degree of support is the key factor for development of a high durability catalyst. Xue [44] et al. compared the ORR activity of Ketjenblack EC-600 as catalyst support with original EC-600 as catalyst and JM60% catalyst after high temperature heat treatment at 1600 °C and 1800 °C. The results showed that the initial particle size of the prepared catalyst ranged from 2.80 to 3.09 nm. After start-stop protocol testing, the particle size of Pt/EC-600 increased from 2.80 to 5.31 nm, and the particle size of Pt/EC-G1800 had the minimum increase in particle size, from 2.99–3.65 nm. Meanwhile, Pt/EC-G1800 catalyst maintains the maximum ORR activity and the best performance after both stress testing protocols. Moreover, the modification of catalyst support is also a common way to improve the electrochemical performance and durability of FCs, such as coating TiO_2_ [45] or synthesizing TiO_x_-C [46], doping Mn or N [47,48,49,50], coating polymeric graphitic carbon nitride [51], and adding silicon oxide [52], etc.

Finally, the preparation of the catalyst faces the problem of mass production. At present, there are many reports on the high performance or durability of the catalyst, but few related to the batch scale-up preparation process [53].

Therefore, it is necessary to prepare high durability catalysts in batch and verify its performance from half-cell to single cell to the stack. In this study, a Pt/graphitic black pearl (GBP) catalyst was synthesized by the formic acid reduction method, which is simple and easy to scale up. The preparation process in this paper can be directly put into mass production. The as-prepared catalyst was characterized by a rotating disk electrode (RDE), and ADT were performed between 0.6–1.0 V and 1.0–1.5 V versus a reversible hydrogen electrode (RHE) (same below, no more annotations), respectively. The 25 cm^2^ Pt/GBP-MEA was prepared, and 130 k cycles ADT with 0.6–1.0 V potential scanning and a 1.5 V high-potential resistance test and a reversal tolerance test were performed, respectively, to evaluate the single-cell performance of the as-prepared catalyst. At last, a three-cell stack was assembled to test the durability with dynamic load cycles. All the above test results showed that the as-prepared Pt/GBP catalyst exhibited better performance than that of Pt/C, including the stability, durability, high-potential resistance and reversal tolerance. The durability of the stack is improved because of the durability and stability of the catalyst. The rapid increase in the hydrogen permeation flow rate may be the main reason for the degradation of FC stack performance. Therefore, Pt/GBP is a valuable and promising catalyst for PEMFC, which can be used as a substitute for classical Pt/C.

## 2. Experimental

### 2.1. Batch Preparation of Pt/GBP Catalyst

Preparation of carbon support: the GBP carbon supports were synthesized by high-temperature heat treatment. The high purity Ar (99.99%, 15 L min^−1^) atmosphere was kept in a high-temperature graphite crucible furnace (XR-SMH-50×100, 196 L, Hunan Xirui Automation Equipment Co., Ltd., Xiangtan, China), the black pearls (BP) 2000 precursors (Suzhou Yi Long Sheng Co., Ltd., Suzhou, China) were mixed with a graphitization catalyst Ni(NO_3_)_2_ (AR, ≥98%), and the weight ratio of carbon to nickel was 20:1. Black Pearls (BP) 2000 is a kind of carbon black with high specific surface area (about 1500 m^2^ g^−1^) and particle size of 15 nm. It has excellent electrical conductivity and is a common support material for fuel cell catalysts. The furnace was heated at different temperatures by an induction coil, including 2400 °C, 2200 °C, 2000 °C, 1800 °C for 1.5 h, and cooled naturally to below 200 °C in an Ar atmosphere. After washing, filtering, and drying, the GBP supports treated at different temperatures were obtained, and the samples were recorded as GBP-2400, GBP-2200, GBP-2000, GBP-1800, respectively.

Preparation of the catalyst: the washed GBP powders were weighed, and then mixed evenly with formic acid (HCOOH, AR, 98%) and ethylene glycol ((CH_2_OH)_2_, AR, 99.5%). After the ultrasonic dispersion for 40 min, the solution was transferred to the reaction kettle and stirred in a nitrogen atmosphere for 40 min. The size of the reaction kettle depended on the amount of catalyst prepared and ensured that the mixing was uniform. The hexahydrate chloroplatinic acid (H_2_PtCl_6_·6H_2_O, AR, 99.95%) aqueous solution (50 wt. %), ethylene glycol and deionized water (Resistivity ≥ 18 MΩ cm) were fed into the reaction kettle, and then stirred in a nitrogen atmosphere for about 40 min and then the temperature was raised to 70 °C. A certain amount of sodium carbonate (Na_2_CO_3_, AR, 99.8%) aqueous solution (0.25 g mL^−1^) was added, and the temperature in the reaction kettle was maintained at 70 °C for 4 h. After the reaction, the mixed solution was filtered in a Brinell funnel with a large amount of deionized water until the filtrate was neutral. The filter cake was transferred into the oven and vacuum dried at 130 °C for 12 h to obtain the catalyst with Pt loading of 60%. Carbon supports treated at different temperatures were used to prepare the corresponding catalysts. Batch preparation can be achieved by increasing the amount of the above materials proportionally, as there are no other factors that inhibit batch preparation. For example, for a 50 L reactor, the catalyst production scale could reach 500–1000 g, theoretically.

### 2.2. Fabrication of MEA and Assembly of FC Stack

**Figure 1:**
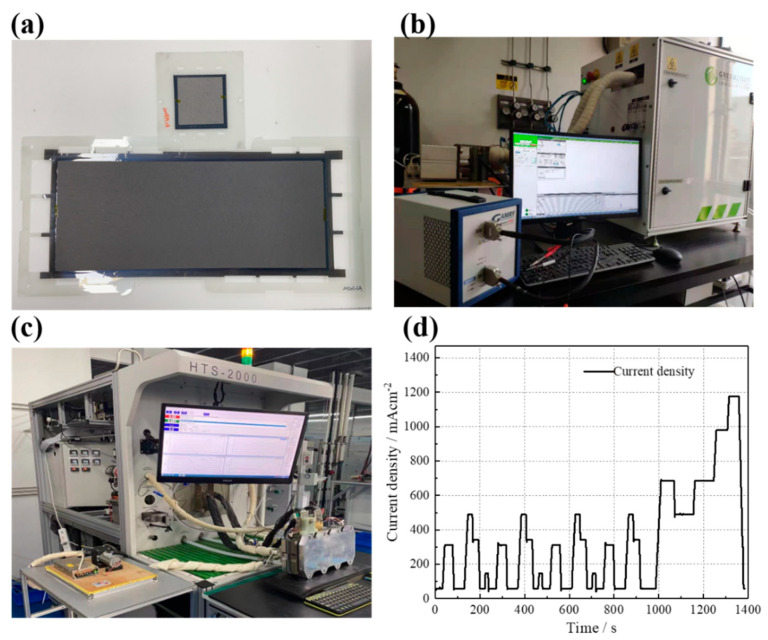
(**a**) 25 cm^2^ MEA, and 340 cm^2^ MEA; (**b**) Greenlight G20 and Gamry reference 3000; (**c**) HTS-2000 (HepHas Energy), and 3-cell stack; (**d**) dynamic load cycle used in 1003-hour durability test.

Fabrication of MEAs: An appropriate amount of as-prepared Pt/GBP or classical Pt/C (JM, 60 wt. %) catalysts were added with a certain amount of Nafion^®^ (5%), which was completely dissolved in the mixture of ultra-pure water and 2-propanol (1:1 in volume) under ultrasonic stirring. The ink was evenly sprayed on the PEM surface (18 μm, Gore Select^®^ M 820, W.L. Gore & Associates, Inc., Newark, DE, USA ) by an automatic coating system (ExactCoat, Sono-Tek Corporation, New York, NY, USA, ). The as-prepared Pt/GBP catalyst and commercial Pt/C (Johnson Matthey JM60% Hispec 9100, Johnson Matthey Technology Centre, London, UK) catalyst were used in the cathode and anode, respectively, while the control samples were obtained using commercial Pt/C for both sides, while the cathode Pt loading was 0.4 mg cm^−2^, and that of the anode was 0.2 mg cm^−2^. The GDL (Avcarb-2240B, Ballard, Vancouver, Canada) was installed and sealed on both sides of the catalytic layer. After hot pressing at 130 °C, MEA with an effective area of 25 cm^2^ and 340 cm^2^ were obtained, respectively, as shown in Figure 1a.

Assembly of FC stack: 3 pieces of MEA with an effective area of 340 cm^2^ were assembled into a stack, and the stack consisted of a front-end plate, back-end plate, collector plates, seal rings, graphite plates and MEAs. As shown in Figure 1c, the length, width and height of the stack were 385, 137 and 85 mm, respectively.

### 2.3. Composition and Structural Characterization of Catalyst

A Hitachi 7700 microscope (Hitachi, Tokyo, Japan) was used to obtain the transmission electron microscope (TEM) images by operating at 100 kV, and higher resolution images of catalyst morphology and structure were obtained at 200 kV by JEM2100F high-resolution transmission electron microscope (HRTEM, Japanese Electronics Co., Ltd., Tokyo, Japan).

The catalyst’s crystal-phase structure was investigated by X-ray diffractometer (XRD). The Cu-Kα rays (0.15406 nm) was used by Bruker D8 Advance Diffractometer to record XRD patterns at 100 mA and 40 kV. The scanning range was 30–90° and the scanning speed was 5° min^−1^.

The carbon support’s graphitization degree was characterized by laser Raman spectroscopy (Invia-reflex, ReniShaw, London, UK). The laser wavelength was 532 nm and 785 nm, and the spectral range was 100–3200 cm^−1^.

The catalyst was characterized by X-ray photoelectron spectroscopy (XSAM800, XPS, Kratos Analytical Ltd., Manchester, UK) before and after the 1003-hour durability test. The Mg-Kα X-ray was used as an excitation source, the photon energy was 1253.6 eV, and the power was 192 W.

### 2.4. Electrochemical Test of Catalyst

The RDE (RDE 710, Pine, North Carolina, USA) and electrochemical workstation (Gamry, reference 3000) were used to investigate the electrochemical performance of the catalyst. The three-electrode system was established in 0.1 M perchloric acid (HClO_4_, AR, 70%) solution at 25 °C. Reversible hydrogen electrode (RHE) was used as a reference electrode (RE), and the Pt wire was used as a counter electrode (CE); the glass carbon electrode coated with catalyst was used as a working electrode (WE). The preparation process of WE was as follows: isopropanol and 5% of the Nafion solution was mixed according to the mass ratio of 30:1 (calculate the dry weight of Nafion), and the mixture was homogenized by ultrasonic for 5 min. A total of 4 mL of the above mixture was poured into a glass vial, 8 mg catalyst was added, and stir vigorously to obtain the catalyst ink. The stirring process lasted for at least 60 min, and the water bath temperature did not exceed 30 °C to prevent catalyst agglomeration caused by high temperatures. A geometric area of 0.196 cm^2^ was used to coat 10 μL of the catalyst ink on a 5 mm diameter glass carbon electrode, at 5 μL each time. After the first coating naturally dried, the second coating was applied to form a working electrode.

Cyclic voltammetry (CV): The scanning rate was 0.05 V s^−1^ (0.05–1.15 V) and the scan was conducted with 30 cycles. The last cycle was recorded as the basis of the ECSA calculation. The test was conducted in HClO_4_ solution (0.1 M) saturated with N_2_ at 25 °C.

Linear sweep voltammetry (LSV): The scan was performed at 0.005 V s^−1^ (0.05–1.15 V), and the rotating speed was 1600 rpm. The test was conducted in HClO_4_ solution (0.1 M) saturated with O_2_ at 25 °C.

Two types of ADT were the 30 k cycles among 0.6–1.0 V and the 10 k cycles among 1.0–1.5 V. The test was conducted in N_2_ saturated HClO_4_ solution (0.1 M) with the sweep rate of 100 mV s^−1^ at 25 °C.

The ECSA (m^2^/g) of the catalyst is calculated according to the electric quantity Q_H_(C/m^2^) obtained by the area integral of the adsorption or desorption peak after deducting the double electric layer area from the measured CV curve. The formula is as follows:

$$\mathrm{ECSA}=Q_{H}/(2.1\times \left[\mathrm{Pt}\right])$$

where [Pt] is the Pt content in the working electrode,2.1 is the adsorption charge constant of hydrogen oxidation on smooth platinum surface per unit area..

Mass activity MA ($\mathrm{mA}/{\mathrm{mg}}_{\mathrm{Pt}}$) is the ORR activity of the catalyst calculated by LSV curve:

$$\mathrm{MA}=j_{k}/\left[\mathrm{Pt}\right]$$

$$\frac{1}{j}=\frac{1}{j_{k}}+\frac{1}{j_{L}}$$

where, J is the apparent current density, j_k_ is the dynamic current density, and j_L_ is the limited current density.

Specific activity is the ORR activity of catalyst per unit active area, which is calculated by:

SA=MA/ECSA

### 2.5. Single-Cell Test of MEA

To evaluate the durability of Pt/GBP and commercial Pt/C catalysts in single cells, the electrochemical performance tests were performed in a standard 25 cm^2^ single cell. The 25 cm^2^ MEA was directly put into the single-cell fixture (provided by Henan Yuqing Power Co., Ltd., Henan, China) for testing.

Single-cell polarization curve test: A Greenlight G20 (Greenlight innovation) FC test bench was used, as shown in Figure 1b. The test conditions were as follows: the gas inlet pressures were 100/80 kPa (G) (An/Ca), the H_2_ and air’ relative humidity were both 80%RH, the stoichiometric ratios were 1.5/2.5 (An/Ca), and the cell temperature was maintained at 75–76 °C.

In the following three types of electrochemical ADT test process, the test conditions were as follows: the single-cell temperature was maintained at 80 °C, the anode and cathode were filled with 200 mL min^−1^ H_2_ and N_2_ with 80%RH, the stoichiometric ratios were 1.7/3.0(An/Ca), and the gas inlet pressures were 120/120 kPa (G) (An/Ca), respectively.

(1) The 0.6–1.0 V potential scanning ADT: Triangle-wave potential cycle scanning was performed with an electrochemical workstation (Gamry, Reference 3000). A total of 130 k cycles were performed between 0.6–1.0 V vs. RHE. After a certain number of cycles were completed, the polarization curve test of MEA was obtained;
(2) Anti-high-potential resistance test: The electrochemical workstation was used to test the single cell with 1.5 V high potential for 1800 s, and the polarization curves and CV curves were measured before and after the test to evaluate the changes of MEA performance;
(3) Reversal tolerance test: A reverse current of 200 mA cm^−2^ from anode to cathode was provided for the single cell by using a DC power supply, and the voltage curve with the time of the single cell from open circuit voltage (OCV) to −2.0 V was recorded. The tolerance of MEA under a reversal condition was investigated. At the same time, polarization curves were measured before and after the test to evaluate the changes of MEA performance.

### 2.6. Durability Test of FC Stack

As shown in Figure 1c, a durability test of the 3-cells stack was carried out on the HTS-2000 (HepHas Energy, Taiwan, China) test bench. The initial polarization curve of the stack was tested after activation and was measured every 100 h during the durability test.

Durability test condition: New European Driving Cycle (NEDC) was a commonly used dynamic cycle, which simulated the process of vehicle acceleration, deceleration and constant speed. It consisted of four repeated low-speed cycles in the city (195 s each) and a 400 s expressway driving cycle. In the original NEDC working conditions, a long OCV time causes excessive degradation of MEA. This study mainly investigated the effect of load dynamic change on FC durability, and therefore, based on NEDC, the OCV time in the cycles was completely removed. The speed of load up/down was 9 A s^−1^. The working condition spectrum of dynamic load cycle was shown in Figure 1d, and the disassembly analysis of each stage is shown in Table 1.

The operating conditions in the above working condition are shown in Table 2, in which the anode pulse drainage frequency and air stoichiometric ratio changed with the dynamic load, the hydrogen/air inlet pressure were constant at 100/80 kPa (G), the water inlet temperature changed with the load between 73–76 °C, the anode was not humidified, and the humidity of the cathode was kept at 45%RH.

## 3. Results and Discussion

### 3.1. Selection of Pt/GBP Catalysts

**Figure 2:**
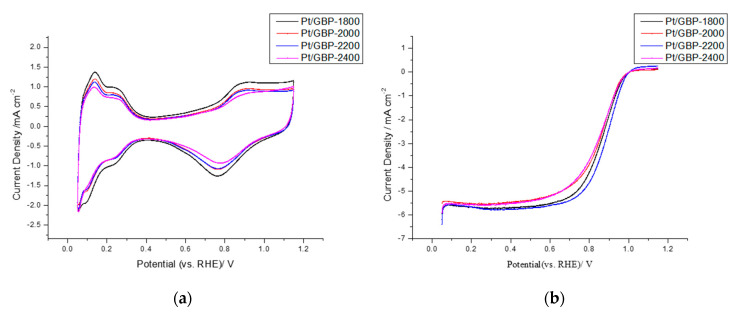
(**a**) CV curves, and (**b**) LSV curves of catalyst prepared by support at different heat treatment temperatures.

For the sake of select suitable carbon supports, the Pt/GBP catalyst was synthesized by carbon supports treated at different temperatures. CV and LSV tests were carried on the self-made catalysts directly (Figure 2), and the corresponding oxygen reduction reaction activity parameters-ECSA, mass activity (MA)and specific activity (SA) estimated at 0.9 V are summarized in Table 3.

It can be seen that with the increase in heat treatment temperature of carbon support, the lattice parameters and particle size of the prepared catalyst gradually increases. In terms of the electrochemical performance, the catalyst ECSA synthesized by carbon supports treated at different temperatures was different, in which Pt/GBP-1800 was the largest, 35.61 m^2^ g_Pt_^−1^. In addition, the MA and SA of different samples were also different. The results showed that the ECSA decreased gradually with the increase of heat treatment temperature. This might be related to the decrease in defect sites of graphitized carbon with the increase in heat treatment temperature, causing the corresponding decrease in platinum particle deposition sites in the process of liquid phase reduction. However, the MA of Pt/GBP-2200 reached 87.57 mA mg_Pt_^−1^, and the SA reached 289.87 μA cm_Pt_^−2^, which indicated that although the reaction active sites reduced correspondingly, the intrinsic catalytic activity per unit mass or unit electrochemical active area was improved. In contrast, when the heat treatment temperature was 2200 °C, the MA and SA of Pt/GBP were the highest. However, compared with the carbon support prepared under the condition of 2200 °C, the corresponding ECSA and MA of 2400 °C are reduced, indicating that the graphitization degree of carbon support was too high under the high temperature, and the defect sites on the surface was excessively reduced, which ultimately affects the adhesion of platinum nanoparticles on the surface of the support, resulting in the reduction of the ECSA. For the same amount of platinum, the reduction in attachment sites will reduce the amount of nucleation of platinum particles, increase the particle size, reduce the utilization rate of platinum, and reduce the intrinsic activity of catalyst particles. Therefore, Pt/GBP-2200 was selected as the catalyst used in this study. For simplicity, Pt/GBP was used in the following text instead of Pt/GBP-2200.

In our previous work [37], the peak of Ni (44°, 52° and 77°) did appear in the XRD test of the carbon support after heat treatment at 1600 °C. The EDX analysis results showed that the content of Ni was less than 1%. However, when the temperature rises again, the peak of Ni disappears completely. We believe that because Ni and C form eutectic structure, their melting point and boiling point will be reduced and can be separated from the carbon support in the form of gas near 2000 °C. In the process of graphitization catalytic, the melting point of Ni(NO_3_)_2_ is 57 °C, and it begins to decompose at about 110 °C, and the boiling point is 137 °C, and it is completely decomposed into NiO, NO_X_, and O_2_ when heated to 300–400 °C. At the same time, due to the reducibility of carbon above 800 °C, it can react with NiO to produce elemental Ni and CO. The metal nanoparticles migrate in the carbon matrix, thus enlarging the original pore size.

$$\mathrm{Ni}{\left({\mathrm{NO}}_{3}\right)}_{2}\to \mathrm{NiO}+\mathrm{NO}+{\mathrm{NO}}_{2}+O_{2}$$

NiO+C→Ni+CO

The boiling point of Ni is 2732 °C, but it is worth noting that the melting point of Ni is 1453 °C, and the evaporation temperature of Ni is 1535 °C. In our experiment, there are three reasons for the disappearance of Ni in the heat treatment process:

(1) Before reaching the boiling point temperature of Ni, the heat treatment temperature used in this experiment has reached its evaporation temperature. In this process, Ni is actually evaporating and decreasing;
(2) The mixing of Ni and carbon matrix makes Ni and C exist in the form of eutectic structure, which lowers the melting and boiling temperature of the mixture. In this experiment, the lowest heat treatment temperature is 1800 °C, and Ni is easy to form in eutectic structure and escape in the form of gas [37];
(3) The mass ratio of Ni to C used in the experiment is 1:20, which means the mass fraction of Ni itself is low. In addition, due to the flow of the inert gas Ar, Ni under the heat treatment conditions of 1800 °C and above is easier to escape and flow out in the form of gas.

### 3.2. Physical Characterizations of Pt/GBP Catalyst

**Figure 3:**
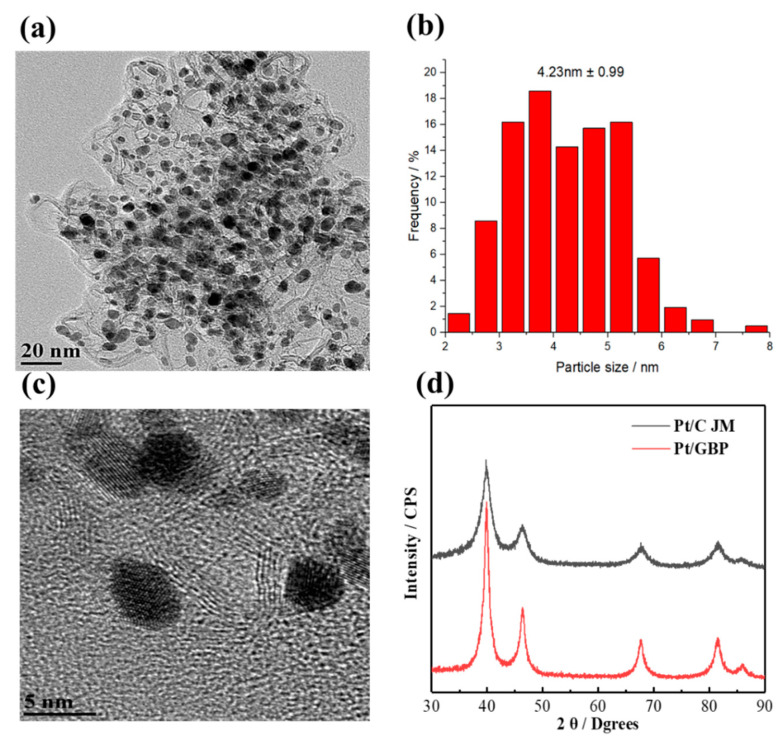
Pt/GBP catalyst: (**a**) the TEM image, (**b**) the histogram of particle size distribution, (**c**) the HRTEM image, and (**d**) the XRD spectrum compared with Pt/C JM catalyst.

In order to investigate the morphology and physical parameters of as-prepared Pt/GBP catalyst, the related characterizations were carried out. The TEM and HRTEM images were shown in Figure 3a,c, respectively. It could be seen that the catalysts are dispersed uniformly without obvious agglomeration, which indicated that the process adopted successfully realized the uniform dispersion of Pt nanoparticles. In addition, Figure 3c showed that the GBP support had many lattice fringes but discontinuous, which represented the formation of the graphitized lamellar structure, but there were defect sites on the surface. As could be seen in Figure 3b, the histogram of particle size distribution showed the particle size mainly concentrated in 2.5–6.0 nm, and the average particle size was 4.23 nm, which was bigger than that of commercial Pt/C (3 nm). Figure 3d showed the comparison of XRD patterns of self-made Pt/GBP and Pt/C catalyst. The results showed that both Pt/GBP and Pt/C catalysts had the typical face centered cubic (fcc) structures, and the main characteristic peaks were 39.76°, 46.24°, 67.47°and 81.71°, correspond to the (111), (200), (220) and (311) crystal planes of Pt nanocrystals, respectively. It was obvious that, the diffraction peak of Pt/GBP was sharper than that of Pt/C, and the full width at half maximum of the Pt/GBP catalyst diffraction peak was significantly smaller than that of commercial Pt/C. This also indicated that the Pt/GBP particle size was larger than that of the latter and was consistent with the above analysis about nanoparticle size. As described in Section 3.1, the increase of platinum particle size is mainly due to the gradual reduction of surface defect sites during the graphitization process of the support at a high temperature, and the reduction of nucleation sites during the liquid phase deposition process, resulting in the growth of platinum particles.

### 3.3. Half-Cell Tests of Pt/GBP and Pt/C Catalyst

**Figure 4:**
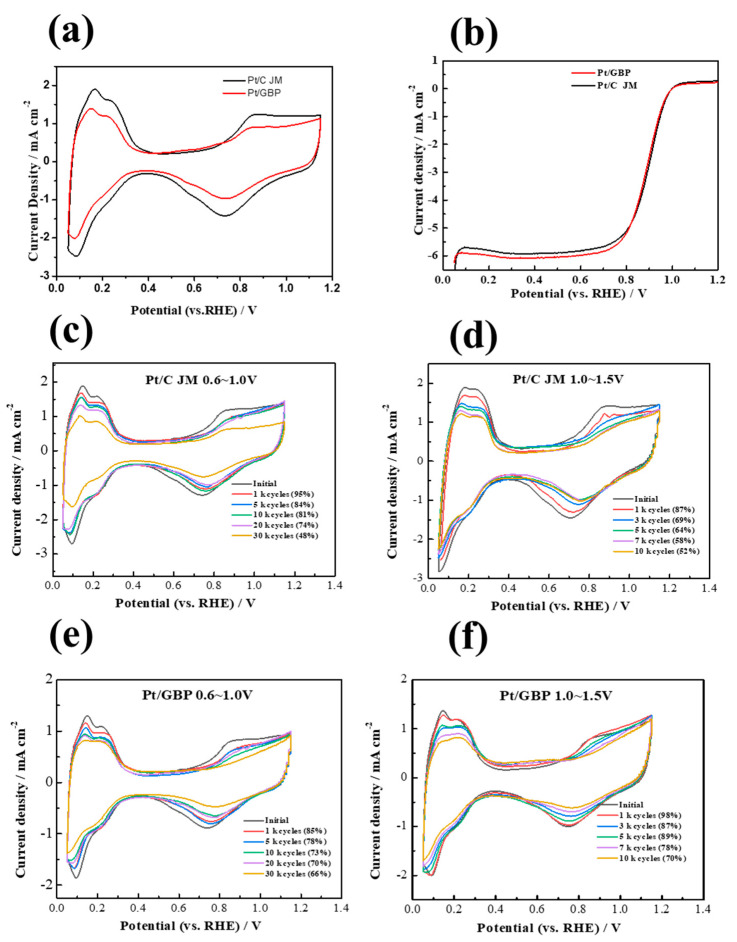
(**a**,**b**) Initial CV, LSV curves of Pt/GBP, Pt/C catalyst; (**c**,**d**) CV curves of Pt/C during 30 k cycles (0.6–1.0 V) and 10 k cycles of ADT (1.0–1.5 V). (**e**,**f**) CV curves of Pt/GBP during 30 k cycles (0.6–1.0 V) and 10 k cycles of ADT (1.0–1.5 V).

CV and LSV curves of self-made Pt/GBP and commercial catalyst Pt/C were shown in Figure 4a,b. The ECSA of the self-made Pt/GBP catalyst was lower than that of commercial Pt/C, which was related to the graphitization structure of the support. Compared with the amorphous carbon of XC-72, the defect sites on the surface of graphitized carbon supports were reduced, which affected the nucleation and deposition of platinum particles. Moreover, the MA of the Pt/GBP catalyst was slightly higher than that of Pt/C, which indicates that the catalytic activity per unit mass was improved; consequently, the SA of Pt/GBP was significantly higher than that of Pt/C. Detailed performance data of the above two catalysts are listed in Table 4.

For the purpose of investigating the electrochemical stability of the self-made catalyst, two kinds of ADT with different severities were carried out at the half-cell level, including low potential 30 k cycles (0.6–1.0 V) and high-potential 10 k cycles (1.0–1.5 V). As shown in Figure 4c,e and Table 5, the ECSA retention rate of Pt/GBP was still 66% after 30 k low potential cycles of 0.6–1.0 V, which was higher than that of commercial Pt/C (48%). Furthermore, it can be observed in Figure 4d,f and Table 6 that the ECSA of Pt/C catalyst decreased by 48% compared with the initial value after 10 k cycles of high potential between 1.0–1.5 V. However, under the same test conditions, ECSA of Pt/GBP only decreased by 30% compared with its initial value under the same test conditions. These results showed that Pt/GBP catalyst exhibited better stability than that of Pt/C in both low- and high-potential cycle. This might be related to the fact that the corrosion resistance of the graphitized carbon support was better than that of amorphous carbon support XC-72 in the FC environment. This may be because after the heat treatment, the original disordered structure is dissolved, and the irregular surface and defects in the support are eliminated. At the same time, some micropores and mesopores are also removed, and the graphitization degree of the carbon support is correspondingly improved. The increase in the graphitization degree of the support is of great help to resist the cyclic corrosion of electric potential in the acidic environment of fuel cell, and avoids the detaching, migration and agglomeration of platinum particles caused by support corrosion.

### 3.4. Single-Cell ADT of Pt/GBP Catalyst

In this paper, the catalyst with 60% Pt loading is mainly considered that the thickness of the prepared catalyst can be appropriately reduced, and the matching with the thickness of the frame and carbon paper is an important process parameter control in the preparation of the catalytic layer. At the same time, the thickness of the catalytic layer is a parameter related to the activation polarization, ohmic polarization and concentration polarization of the single cell. Its structural design is also particularly important to improve the performance and reliability of the electrode. Properly thinning the thickness of the catalytic layer can significantly reduce the influence of ohmic polarization and mass transfer polarization, so as to improve the ultimate current density of the electrode.

**Figure 5:**
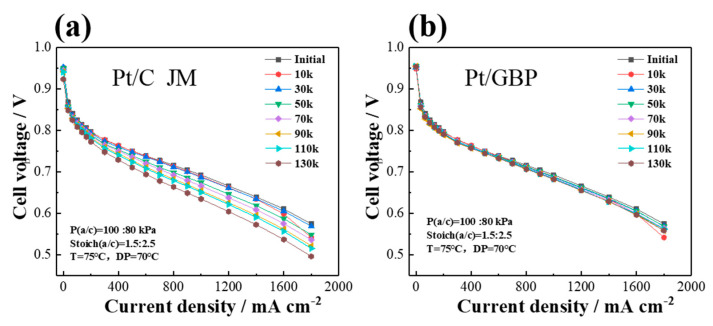
Polarization curves of MEAs with (**a**) commercial Pt/C and (**b**) Pt/GBP catalyst during 130 k cycles ADT between 0.6-1.0 V.

**Figure 6:**
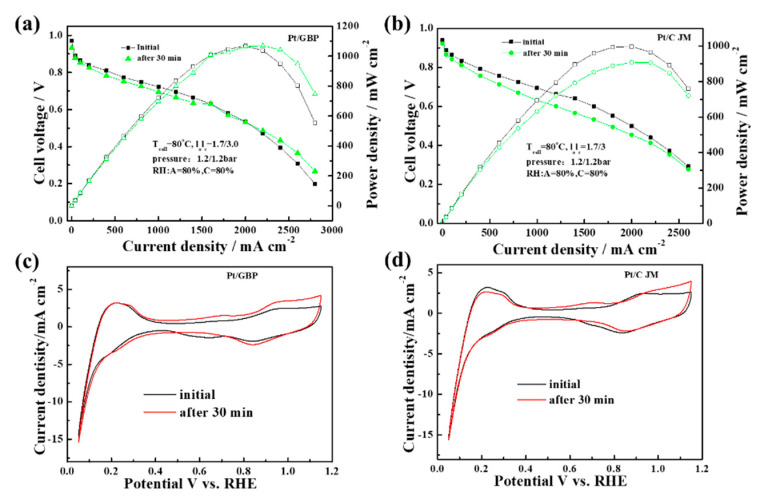
(**a**,**c**) The polarization curves and CV curves of Pt/GBP before and after ADT at 1.5 V for 1800 s; and (**b**,**d**) The polarization curves and CV curves of commercial Pt/C before and after ADT at 1.5 V for 1800 s.

**Figure 7:**
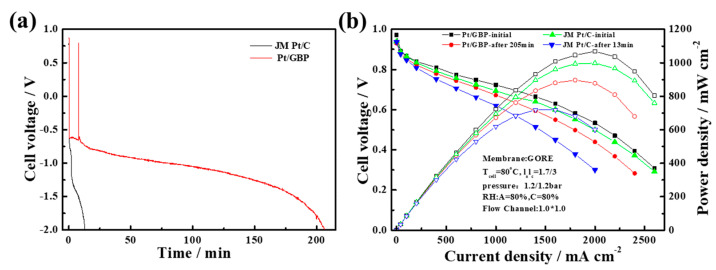
(**a**) Voltage reversal tests of MEAs fabricated with Pt/C and Pt/GBP and (**b**) polarization curves of MEAs before and after reversal tolerance test.

In order to test the durability of catalyst in single cells, three types of ADT were carried out with MEA:

(1) 0.6–1.0 V cycles test: The changes of the polarization performance of commercial Pt/C-MEA and Pt/GBP-MEA during 130 k cycles of 0.6–1.0 V are shown in Figure 5. The commercial Pt/C-MEA showed a significant performance degradation as shown in Figure 5a, with a voltage loss up to 58 mV@ 1000 mA cm^−2^, and the performance degradation was more serious at high current density. However, after 130 k cycles of 0.6–1.0 V, the Pt/GBP-MEA in Figure 5b showed almost no performance loss, only 10 mV@1000 mA cm^−2^. The results indicated that the MEA fabricated with Pt/GBP had stronger potential-cycle resistance in the range of 0.6–1.0 V, and showed better durability than Pt/C;
(2) Anti-high penitential (1.5 V) test: In order to simulate the state of high potential caused by FC stack start-up/shut-down and local reversal, ADT with a high potential of 1.5 V was carried out. The polarization curves and CV curves of MEAs fabricated with Pt/GBP and Pt/C before and after ADT are illustrated in Figure 6. At 1000 mA cm^−2^, the initial performance for Pt/GBP and Pt/C were 0.724 V and 0.693 V, respectively. After 1800 s of the sustained high-potential scanning of 1.5 V, the voltages of both decreased to 0.695 V and 0.621 V at 1000 mA cm^−2^, and decay rates were 4% and 10.3%, respectively. In fact, as could be seen from the polarization curves in Figure 6a, the performance of the PGB catalyst was still increasing at high current density. Figure 6c,d showed the comparison of CV curves of single cells before and after ADT. It could be seen that ECSA of Pt/GBP-MEA had almost no change after 1800 s at high potential of 1.5 V, while Pt/C catalyst decreased significantly. The results indicated that the as-prepared Pt/GBP catalyst had excellent resistance and longer lifetime to high potential, implying that high graphitized support had strong corrosion resistance under high-potential conditions;
(3) Reversal tolerance test: In order to investigate the reversal tolerance of MEA, the single-cell reversal tolerance tests were carried out. Figure 7a shows the voltage–time curves based on MEA fabricated with Pt/C and Pt/BPG catalysts during the test. The results demonstrated that the voltage of Pt/C-MEA decreased rapidly and only survives for about 13 min, however, Pt/GBP-MEA decreased slowly from 0.6 to −2.0 V, and the survival time reached 205 min, indicating that the Pt/GBP-MEA had better reversal tolerance. Figure 7b showed the polarization curves of MEA fabricated with different catalysts before and after reversal tolerance test. The Pt/GBP-MEA had a smaller voltage loss (51 mV) than Pt/C-MEA (74 mV) @1000 mA cm^−2^, which also could be ascribed to the better corrosion resistance of GBP support.

### 3.5. Durability Test of Pt/GBP Catalyst in PEMFC Stack

**Figure 8:**
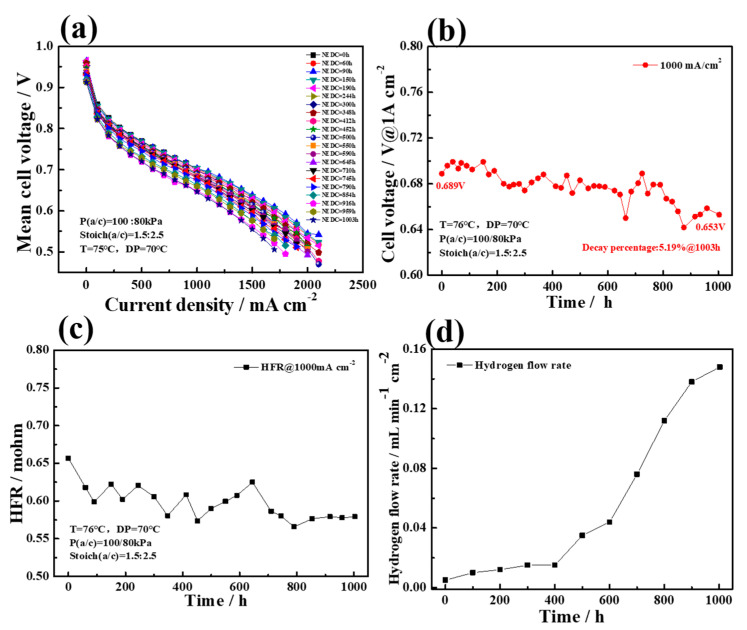
(**a**) Polarization curves during durability test, (**b**) the change of mean cell voltage, (**c**) the change of HFR, and (**d**) the change of H_2_ crossover flow rate during the durability test.

For the purpose of further evaluating the application of the PEMFC stack in practical conditions, the 1003-hour durability test was performed, and the actual driving conditions were simulated by the dynamic load cycles. Figure 8a showed the variation of the polarization curves during the stack durability test. Obviously, the stack performance declines gradually over time. The average voltage variation at 1000 mA cm^−2^ was shown in Figure 8b. It could be seen that in the first 800 h, the performance of the stack was relatively stable, the average cell voltage doesn’t change too much, the degradation rate was only 9 μV h^−1^, and the degradation percentage was 1.4%. However, in the last 203 h, the average cell voltage of the stack decreased rapidly, the total degradation rate reached to 36 μV h^−1^ at 1003 h, and the degradation percentage reached to 5.19%. This results implied that the stack had a lower average voltage decay rate than that of our previous study (14.4% after 420 h at 800 mA cm^−2^ [54]), which indicated that the Pt/GBP catalyst had better durability.

The high-frequency impedance (HFR) was generally defined as the sum of the ohmic impedance of all the components, and the interface contact resistance between the components in the FC stack. Among them, the ion impedance of PEM was the main part of HFR and was significantly affected by the water content in the membrane. Figure 8c showed the change of HFR in the stack over time at 1000 mA cm^−2^. It was worth noting that, the HFR generally decreased over time. First of all, the state of inter-layer connection was getting better. With the progresses of the durability test, the connection of the catalyst layer and microporous layer was closer under the effect of assembly force. In addition, the deterioration of hydrophobicity of the catalyst or GDL may have led to an increase in liquid water content in MEA, which could have effectively increased the ion conductivity of PEM, resulting in the increase in ionic conductivity and the decrease of HFR.

Figure 8d showed the variation curve of hydrogen permeation flow rate during the 1003-hour durability test. The hydrogen permeation rate increased slowly at the beginning, reaching 0.035 mL min^−1^ cm^−2^ at 500 h. Then it increased sharply, and reaching 0.148 mL min^−1^ cm^−2^ at 1003 h. This would increase the leakage polarization and reduced the FC output voltage directly. The OCV decreased from the initial 0.958 V to 0.912 V with a decrease of 46 mV after 1003 h durability test. There were two possible reasons for the increase in the hydrogen permeation flow rate. One could be ascribed to the gradual failure of the sealing frame during operation, leading to hydrogen leakage directly from the failure place. In addition, chemical degradation of PEM caused by the dynamic load cycle and external environment, thinner membrane and poor gas resistance also lead to the increase of hydrogen permeation flow rate. Therefore, the increase in the hydrogen permeation flow rate caused by the sealing frame failure or the PEM degradation may be the main reasons for the rapid increase of degradation rate of the stack.

### 3.6. Post-Morphology Characterizations of Pt/GBP Catalyst

**Figure 9:**
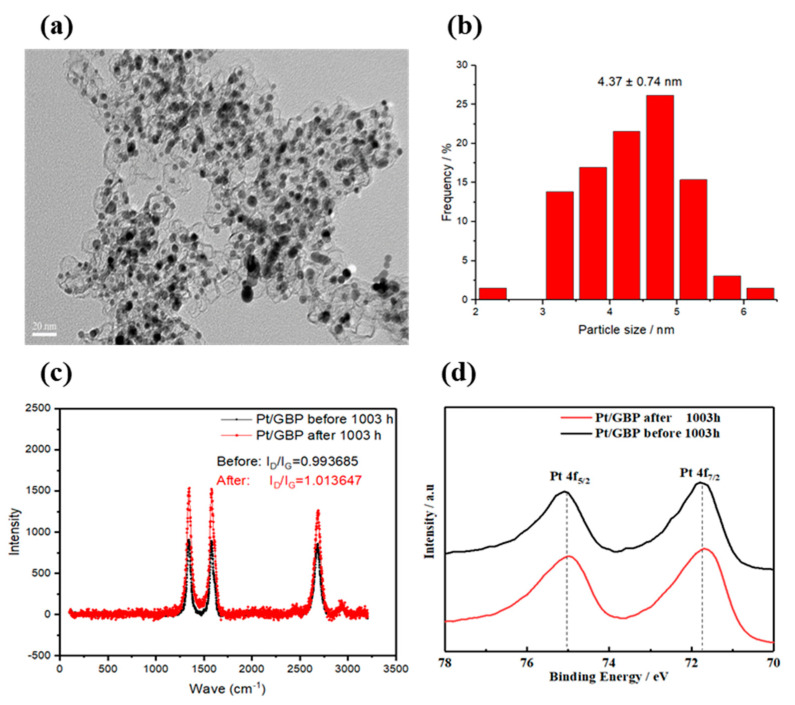
(**a**)TEM images of the Pt/GBP catalysts in cathode after 1003 h test; (**b**) particle size distribution after durability test; (**c**) Laman and (**d**) XPS spectrum before and after durability test.

To further clarify the change of catalyst, the morphology and characteristic parameters of Pt/GBP cathode catalyst after the 1003-hour durability test were characterized. The TEM images of Pt/GBP catalyst after the 1003-hour test are illustrated in Figure 9a. After 1003 h of dynamic loading cycles, the overall morphology did not change significantly, and the Pt nanoparticles were still evenly dispersed on the GBP support. According to the histogram of particle size distribution in Figure 9b, the particle size of nanoparticles remained about 4 nm. These results implied that enhanced graphitization degree of carbon support GBP could effectively inhibit the agglomeration of nanoparticles, and improve its corrosion resistance, which is helpful to improve the durability of Pt/GBP catalyst under the actual operating condition. Figure 9c, d showed the Laman spectrum and XPS of Pt/GBP catalyst after durability test, which had no significant changes compared with those before the test. Figure 9c showed the Raman spectra of Pt/GBP before and after durability test. The intensity ratio of D peak (1344 cm^−1^) and G peak (1580 cm^−1^), i.e., I_D_/I_G_ indicated the ordered degree of the structure. It can be seen that there was no significant change before and after the durability test, indicating that the graphitization degree of carbon support changes litter. In addition, Figure 9d showed the Pt 4f spectrum of Pt/GBP catalyst before and after the durability test of 1003 h. Negligible changes could be observed in binding energy of the strong peaks Pt 4f_5/2_ and Pt 4f_7/2_ after ADT of 1003 h, which implied robust structure of the Pt/GBP catalyst.

## 4. Conclusions

In this study, the Pt/GBP catalyst was synthesized in batch by the formic acid reduction method. The average particle size of the synthesized Pt particles was 4.2 nm, and which were uniformly dispersed on the supports. The ECSA of self-made Pt/GBP catalyst was lower than that of commercial Pt/C, but the MA of Pt/GBP catalyst was slightly higher than that of Pt/C, and consequently, the SA of Pt/GBP was significantly higher than that of Pt/C. Through the verifications and characterizations of the half-cell, single-cell and stack, the main outcomes of this study could be summarized as:

(1) The half-cell test shows that Pt/GBP catalyst had good stability and durability. After 30 k cycles between 0.6–1.0 V, the ECSA retention rate of Pt/GBP was 66%, which was higher than that of Pt/C (48%). After 10 k cycles between 1.0–1.5 V, the ECSA of Pt/GBP decreased by only 30% compared with the initial value, while that decreased by 48% for Pt/C under the same conditions;
(2) After 130 k triangle-wave potential cycle scanning, the voltage loss of Pt/GBP-MEA was only 13 mV, while that of Pt/C under the same condition was 50 mV at 1000 mA cm^−2^. After 1800 s of potentiostatic ADT at 1.5 V, the voltage loss of the Pt/GBP-MEA was 28 mV and the decay rate was 3.8% at 1000 mA cm^−2^, and it was lower than the 73 mV and 10.5% of commercial Pt/C. The reversal tolerance time of the Pt/GBP-MEA was 205 min, which was much longer than 13 min of commercial Pt/C;
(3) A 3-cell stack was assembled for a durability test under dynamic cycle conditions of 1003 h. Before 800 h, the stack performance was stable, and the performance degradation rate was 1.4% and 9 μV h^−1^ at 1000 mA cm^−2^. However, in the last 203 h, the stack performance reduces rapidly. After 1003 h of durability test, and the degradation percentage reached to 5.19% and 36 μV h^−1^ at 1000 mA cm^−2^. The sealing frame failure or the PEM degradation and the corresponding increase in hydrogen permeation flow rate, may be the main reasons for the rapid increase in the stack degradation rate after 800 h.

This study shows that the self-made Pt/GBP catalyst has high catalytic activity, stability and durability, especially under harsh conditions, and has great commercial application prospects. In the subsequent experimental design, it is necessary to carry out longer durability test with Pt/GBP catalyst, the precise characterization of the carbon support, the changes of each layer and the interlayer interface of MEA, as well as the migration and deposition effects of Pt nanoparticles and mitigation strategies need to be studied in detail. The reasons for the increase in the hydrogen permeation flow rate need to be analyzed in detail, and corresponding experiments should be designed and verification. The material properties of Pt nanoparticles, Nafion^®^ ionomer and GBP support before and after durability test need to be quantitatively characterized. This work will be very meaningful and will help us to understand the various decay of Pt/GBP catalysts under working conditions, to achieve DOE’s durability goals as soon as possible, and effectively promote the commercial application of FC vehicles.

## Figures and Tables

**Table 1:** The disassembly analysis of each stage of dynamic load cycle.

Potential (V)	Current Density (mA cm^−2^)	Last Time (s)	Proportion of Time (%)	Actual Status
OCV	0	0	0%	Start/stop
0.851	59	417	35%	Idle
0.797	147	52		
0.759	312	140	40%	Medium speed
0.752	341	100		
0.713	488	162		
0.675	685	143		
0.630	979	50	7%	High speed
0.600	1176	44		
0.8–0.6	59–1176	249	18%	Accelerate and decelerate (the speed of load up/down is 9 A s^−1^)

**Table 2:** Operating conditions in the dynamic load cycle.

Current Density (mA cm^−2^)	Anode Pulse Drainage Frequency (s)	Air Stoichiometric Ratio	Inlet Pressure of Gases (kPa(G))		Inlet Temperature of Water (°C)	Inlet Humidity of Gases (%RH)	
			An	Ca		An	Ca
59–312	10/0.1	3.5–3	100	80	73	\	45
312–685	7/0.1	3.5–2.5–3			76		
685–979	5/0.1	2.2			76		
979–1176	5/0.1	2			73		

**Table 3:** The characteristics and electrochemical performance comparison of as-prepared Pt/GBP at different temperatures.

Catalysts	Lattice Parameters Å	Particle Size nm	ECSA (m^2^ g_Pt_^−1^)	MA (mA mg_Pt_^−1^)	SA (μA cm_Pt_^−2^)
Pt/GBP-2400	3.920	5.34	28.74	68.77	239.28
Pt/GBP-2200	3.916	4.07	30.21	87.57	289.87
Pt/GBP-2000	3.913	3.94	31.36	69.93	222.99
Pt/GBP-1800	3.912	3.87	35.61	72.54	203.71

**Table 4:** Performance comparison of self-made Pt/GBP and commercial Pt/C.

Catalysts	ECSA (m^2^ g_Pt_^−1^)	MA (mA mg_Pt_^−1^)	SA (μA cm_Pt_^−2^)
Pt/GBP 60%	30.21	87.57	289.87
Pt/C(JM) 60%	52.24	81.36	155.74

**Table 5:** The ECSA retention rate of Pt/C and Pt/GBP during 30 k cycles (0.6–1.0 V).

	The Initial ECSA (m^2^ g_Pt_^−1^)	The ECSA and Its Retention Rate after 10 k Cycles (m^2^ g_Pt_^−1^)	The ECSA and Its Retention Rate after 20 k Cycles (m^2^ g_Pt_^−1^)	The ECSA and Its Retention Rate after 30 k Cycles (m^2^ g_Pt_^−1^)
Pt/C	53.44	43.29; 81%	39.55; 74%	25.65; 48%
Pt/GBP	30.89	22.55; 73%	21.62; 70%	20.39; 66%

**Table 6:** The ECSA retention rate of Pt/C and Pt/GBP during 30 k cycles (1.0–1.5 V).

	The Initial ECSA (m^2^ g_Pt_^−1^)	The ECSA and Its Retention Rate after 3 k Cycles (m^2^ g_Pt_^−1^)	The ECSA and Its Retention Rate after 7 k Cycles (m^2^ g_Pt_^−1^)	The ECSA and Its Retention Rate after 10 k Cycles (m^2^ g_Pt_^−1^)
Pt/C	52.64	36.32; 69%	30.53; 58%	27.37; 52%
Pt/GBP	31.22	27.16; 87%	24.35; 78%	21.85; 70%

## Data Availability

Not applicable.

## References

[1] Ji Y., Cho Y.I., Jeon Y., Lee C., Park D.-H., Shul Y.-G. (2017). Design of active Pt on TiO_2_ based nanofibrous cathode for superior PEMFC performance and durability at high temperature. Appl. Catal. B Environ..

[2] Sakthivel M., Drillet J.-F. (2018). An extensive study about influence of the carbon support morphology on Pt activity and stability for oxygen reduction reaction. Appl. Catal. B Environ..

[3] Sharaf O.Z., Orhan M.F. (2014). An overview of fuel cell technology: Fundamentals and applications. Renew. Sustain. Energy Rev..

[4] Papageorgopoulos D. (2019). U.S. DOE Annual Merit Review and Peer Evaluation, Fuel Cell R&D Overview.

[5] Borup R., Meyers J., Pivovar B., Kim Y.S., Mukundan R., Garland N., Myers D., Wilson M., Garzon F., Wood D. (2007). Scientific Aspects of Polymer Electrolyte Fuel Cell Durability and Degradation. Chem. Rev..

[6] Yazici M.S., Azder M.A., Salihoglu O., San F.G.B. (2018). Ultralow Pt loading on CVD graphene for acid electrolytes and PEM fuel cells. Int. J. Hydrogen Energy.

[7] Jahnke T., Futter G., Latz A., Malkow T., Papakonstantinou G., Tsotridis G., Schott P., Gérard M., Quinaud M., Quiroga M. (2016). Performance and degradation of Proton Exchange Membrane Fuel Cells: State of the art in modeling from atomistic to system scale. J. Power Sources.

[8] Bharti A., Cheruvally G., Muliankeezhu S. (2017). Microwave assisted, facile synthesis of Pt/CNT catalyst for proton exchange membrane fuel cell application. Int. J. Hydrogen Energy.

[9] Massaglia G., Margaria V., Sacco A., Castellino M., Chiodoni A., Pirri F.C., Quaglio M. (2019). N-doped carbon nanofibers as catalyst layer at cathode in single chamber Microbial Fuel Cells. Int. J. Hydrogen Energy.

[10] Wang K., Chen H., Zhang X., Tong Y., Song S., Tsiakaras P., Wang Y. (2020). Iron oxide@graphitic carbon core-shell nanoparticles embedded in ordered mesoporous N-doped carbon matrix as an efficient cathode catalyst for PEMFC. Appl. Catal. B Environ..

[11] Chen L., Liu X., Zheng L., Li Y., Guo X., Wan X., Liu Q., Shang J., Shui J. (2019). Insights into the role of active site density in the fuel cell performance of Co-N-C catalysts. Appl. Catal. B Environ..

[12] Yang X., Wang Y., Zhang G., Du L., Yang L., Markiewicz M., Choi J.-Y., Chenitz R., Sun S. (2020). SiO_2_-Fe/N/C catalyst with enhanced mass transport in PEM fuel cells. Appl. Catal. B Environ..

[13] García Á., Retuerto M., Dominguez C., Pascual L., Ferrer P., Gianolio D., Serrano A., Aßmann P., Sanchez D.G., Peña M.A. (2020). Fe doped porous triazine as efficient electrocatalysts for the oxygen reduction reaction in acid electrolyte. Appl. Catal. B Environ..

[14] Chung S., Ham K., Kang S., Ju H., Lee J. (2020). Enhanced corrosion tolerance and highly durable ORR activity by low Pt electrocatalyst on unique pore structured CNF in PEM fuel cell. Electrochim. Acta.

[15] Zhang L., Zhao Y., Banis M.N., Adair K., Song Z., Yang L., Markiewicz M., Li J., Wang S., Li R. (2019). Rational design of porous structures via molecular layer deposition as an effective stabilizer for enhancing Pt ORR performance. Nano Energy.

[16] Yusof M., Jalil A., Ahmad A., Triwahyono S., Othman M., Abdullah T., Firmansyah M.L., Setiabudi H.D., Johari A., Nabgan W. (2019). Effect of Pt–Pd/C coupled catalyst loading and polybenzimidazole ionomer binder on oxygen reduction reaction in high-temperature PEMFC. Int. J. Hydrogen Energy.

[17] Meier J.C., Galeano C., Katsounaros I., Witte J., Bongard H., Topalov A.A., Baldizzone C., Mezzavilla S., Schüth F., Mayrhofer K.J.J. (2014). Design criteria for stable Pt/C fuel cell catalysts. Beilstein, J. Nanotechnol..

[18] Meier J.C., Galeano C., Katsounaros I., Topalov A.A., Kostka A., Schüth F., Mayrhofer K.J.J. (2012). Degradation Mechanisms of Pt/C Fuel Cell Catalysts under Simulated Start–Stop Conditions. ACS Catal..

[19] Bogar M., Yakovlev Y., Sandbeck D.J.S., Cherevko S., Matolínová I., Amenitsch H., Khalakhan I. (2021). Interplay Among Dealloying, Ostwald Ripening, and Coalescence in PtXNi100–X Bimetallic Alloys under Fuel-Cell-Related Conditions. ACS Catal..

[20] Martens I., Chattot R., Drnec J. (2021). Decoupling catalyst aggregation, ripening, and coalescence processes inside operating fuel cells. J. Power Sources.

[21] Beermann V., Gocyla M., Willinger E., Rudi S., Heggen M., Dunin-Borkowski R.E., Willinger M.-G., Strasser P. (2016). Rh-Doped Pt–Ni Octahedral Nanoparticles: Understanding the Correlation between Elemental Distribution, Oxygen Reduction Reaction, and Shape Stability. Nano Lett..

[22] Esfahani R.A.M., Fruehwald H., Laschuk N.O., Sullivan M.T., Egan J.G., Ebralidze I., Zenkina O.V., Easton E.B. (2020). A highly durable N-enriched titanium nanotube suboxide fuel cell catalyst support. Appl. Catal. B Environ..

[23] Lobato J., Zamora H., Plaza J., Cañizares P., Rodrigo M.A. (2016). Enhancement of high temperature PEMFC stability using catalysts based on Pt supported on SiC based materials. Appl. Catal. B Environ..

[24] Choi I., Lee H., Lee K.G., Ahn S.H., Lee S.J., Kim H.-J., Lee H.-N., Kwon O.J. (2015). Characterization of self-humidifying ability of SiO_2_ -supported Pt catalyst under low humidity in PEMFC. Appl. Catal. B Environ..

[25] Stassi A., Gatto I., Baglio V., Passalacqua E., Arico’ A.S. (2013). Oxide-supported PtCo alloy catalyst for intermediate temperature polymer electrolyte fuel cells. Appl. Catal. B Environ..

[26] Park C., Lee E., Lee G., Tak Y. (2020). Superior durability and stability of Pt electrocatalyst on N-doped graphene-TiO_2_ hybrid material for oxygen reduction reaction and polymer electrolyte membrane fuel cells. Appl. Catal. B Environ..

[27] Gasteiger H.A., Kocha S.S., Sompalli B., Wagner F.T. (2005). Activity benchmarks and requirements for Pt, Pt-alloy, and non-Pt oxygen reduction catalysts for PEMFCs. Appl. Catal. B Environ..

[28] Zhang Y., Wang N., Jia N., Wang J., Sun J., Shi F., Liu Z., Jiang R. (2019). A Low-Cost and Facile Method for the Preparation of Fe-N/C-Based Hybrids with Superior Catalytic Performance toward Oxygen Reduction Reaction. Adv. Mater. Interfaces.

[29] Zheng Y., He F., Wu J., Ma D., Fan H., Zhu S., Li X., Lu Y., Liu Q., Hu X. (2019). Nitrogen-Doped Carbon Nanotube–Graphene Frameworks with Encapsulated Fe/Fe_3_N Nanoparticles as Catalysts for Oxygen Reduction. ACS Appl. Nano Mater..

[30] Zhang X., Liu R., Zang Y., Liu G., Wang G., Zhang Y., Zhang H., Zhao H. (2016). Co/CoO nanoparticles immobilized on Co–N-doped carbon as trifunctional electrocatalysts for oxygen reduction, oxygen evolution and hydrogen evolution reactions. Chem. Commun..

[31] Xiao Y.-X., Ying J., Tian G., Tao Y., Wei H., Fan S.-Y., Sun Z.-H., Zou W.-J., Hu J., Chang G.-G. (2019). Highly dispersed PtPd on graphitic nanofibers and its heavy d-π effect. Appl. Catal. B Environ..

[32] Li H., Huang Y., Zhou H., Yang W., Li M., Huang Z., Fu C., Kuang Y. (2017). One step in-situ synthesis of Co@N, S co-doped CNTs composite with excellent HER and ORR bi-functional electrocatalytic performances. Electrochim. Acta.

[33] Hernández-Fernández P., Montiel M., Ocón P., de la Fuente J.G., García-Rodríguez S., Rojas S., Fierro J. (2010). Functionalization of multi-walled carbon nanotubes and application as supports for electrocatalysts in proton-exchange membrane fuel cell. Appl. Catal. B Environ..

[34] Tzorbatzoglou F., Brouzgou A., Tsiakaras P. (2015). Electrocatalytic activity of Vulcan-XC-72 supported Pd, Rh and PdxRhy toward HOR and ORR. Appl. Catal. B Environ..

[35] Siller-Ceniceros A., Sánchez-Castro M., Morales D., Torres-Lubian J.R., G. E.M., Rodríguez-Varela J. (2017). Innovative functionalization of Vulcan XC-72 with Ru organometallic complex: Significant enhancement in catalytic activity of Pt/C electrocatalyst for the methanol oxidation reaction (MOR). Appl. Catal. B Environ..

[36] Wang X., Hsing I.-M., Yue P. (2001). Electrochemical characterization of binary carbon supported electrode in polymer electrolyte fuel cells. J. Power Sources.

[37] Wang J., Xue Q., Li B., Yang D., Lv H., Xiao Q., Ming P., Wei X., Zhang C. (2020). Preparation of a Graphitized-Carbon-Supported PtNi Octahedral Catalyst and Application in a Proton-Exchange Membrane Fuel Cell. ACS Appl. Mater. Interfaces.

[38] Zhao W., Ye Y., Jiang W.-J., Li J., Tang H., Hu J.-S., Du L., Cui Z., Liao S. (2020). Mesoporous carbon confined intermetallic nanoparticles as highly durable electrocatalysts for the oxygen reduction reaction. J. Mater. Chem. A.

[39] Lee H.-B.-R., Baeck S.H., Jaramillo T., Bent S.F. (2013). Growth of Pt Nanowires by Atomic Layer Deposition on Highly Ordered Pyrolytic Graphite. Nano Lett..

[40] Roen L.M., Paik C.H., Jarvi T.D. (2004). Electrocatalytic Corrosion of Carbon Support in PEMFC Cathodes. Electrochem. Solid-State Lett..

[41] Shao Y., Yin G., Gao Y. (2007). Understanding and approaches for the durability issues of Pt-based catalysts for PEM fuel cell. J. Power Sources.

[42] Lamibrac A., Maranzana G., Dillet J., Lottin O., Didierjean S., Durst J., Dubau L., Maillard F., Chatenet M. (2012). Local Degradations Resulting from Repeated Start-ups and Shut-downs in Proton Exchange Membrane Fuel Cell (PEMFC). Energy Procedia.

[43] Zhao X., Hayashi A., Noda Z., Kimijima K., Yagi I., Sasaki K. (2013). Evaluation of change in nanostructure through the heat treatment of carbon materials and their durability for the start/stop operation of polymer electrolyte fuel cells. Electrochim. Acta.

[44] Xue Q., Huang J.-B., Yang D.-J., Li B., Zhang C.-M. (2021). Enhanced PEMFC durability with graphitized carbon black cathode catalyst supports under accelerated stress testing. RSC Adv..

[45] Shi W., Park A.-H., Xu S., Yoo P.J., Kwon Y.-U. (2021). Continuous and conformal thin TiO_2_-coating on carbon support makes Pd nanoparticles highly efficient and durable electrocatalyst. Appl. Catal. B Environ..

[46] Esfahani R.A.M., Videla A.H.M., Vankova S., Specchia S. (2015). Stable and methanol tolerant Pt/TiOx-C electrocatalysts for the oxygen reduction reaction. Int. J. Hydrogen Energy.

[47] Liu K., Qiao Z., Hwang S., Liu Z., Zhang H., Su D., Xu H., Wu G., Wang G. (2019). Mn- and N- doped carbon as promising catalysts for oxygen reduction reaction: Theoretical prediction and experimental validation. Appl. Catal. B Environ..

[48] Ratso S., Kruusenberg I., Joost U., Saar R., Tammeveski K. (2016). Enhanced oxygen reduction reaction activity of nitrogen-doped graphene/multi-walled carbon nanotube catalysts in alkaline media. Int. J. Hydrogen Energy.

[49] He D., Jiang Y., Lv H., Pan M., Mu S. (2013). Nitrogen-doped reduced graphene oxide supports for noble metal catalysts with greatly enhanced activity and stability. Appl. Catal. B Environ..

[50] Chen Y., Wang J., Liu H., Li R., Sun X., Ye S., Knights S. (2009). Enhanced stability of Pt electrocatalysts by nitrogen doping in CNTs for PEM fuel cells. Electrochem. Commun..

[51] Lee I.H., Cho J., Chae K.H., Cho M.K., Jung J., Cho J., Lee H.J., Ham H.C., Kim J.Y. (2018). Polymeric graphitic carbon nitride nanosheet-coated amorphous carbon supports for enhanced fuel cell electrode performance and stability. Appl. Catal. B Environ..

[52] Wu Y.-N., Liao S.-J., Zeng J.-H. (2011). Investigating the addition of silicon oxide to carbon: Effects of amount and heat treatment on anti-aggregation and electrochemical performance of Pt catalysts. J. Power Sources.

[53] Choi J., Jang J.-H., Roh C.-W., Yang S., Kim J., Lim J., Yoo S.J., Lee H. (2018). Gram-scale synthesis of highly active and durable octahedral PtNi nanoparticle catalysts for proton exchange membrane fuel cell. Appl. Catal. B Environ..

[54] Li B., Higgins D.C., Xiao Q., Yang D., Zhng C., Cai M., Chen Z., Ma J. (2015). The durability of carbon supported Pt nanowire as novel cathode catalyst for a 1.5 kW PEMFC stack. Appl. Catal. B Environ..

